# Patient satisfaction with quality of care of a multidisciplinary thrombosis service – a cross sectional survey

**DOI:** 10.1186/s12913-022-08089-w

**Published:** 2022-05-23

**Authors:** Stephanie W. Young, Kwadwo O. Bonsu, Tiffany Lee, Hai V. Nguyen, Rufaro S. Chitsike

**Affiliations:** 1 School of Pharmacy, Memorial University of Newfoundland, 300 Prince Philip Drive, St John’s, NL A1B 3V6 Canada; 2 Pharmacy Program, Eastern Region Health Authority, 300 Prince Philip Drive, St John’s, NL A1B 3V6 Canada; 3 Division of Hematology, Eastern Region Health Authority, 300 Prince Philip Drive, St. John’s, NL A1B 3V6 Canada; 4 Division of Medicine (Hematology), Memorial University of Newfoundland, 300 Prince Philip Drive, St. John’s, NL A1B 3V6 Canada

**Keywords:** Patient satisfaction, Patient-centered care, Anticoagulation therapy, Thrombosis care, Multidisciplinary team

## Abstract

**Background:**

In October 2017 we opened a multidisciplinary Adult Outpatient Thrombosis Service (Thrombosis Service) in a regional health authority servicing over 300 000 people. The Thrombosis Service is a comprehensive thrombosis and anticoagulation management program with unique, interrelated clinics providing a broad spectrum of care for this patient group. Evaluation of patient satisfaction with this new model of patient care is an important quality measurement.

**Methods:**

We conducted a cross-sectional survey of patients who attended the Thrombosis Service between October 2017 and May 2019. We measured patient satisfaction with the seven-item Short Assessment of Patient Satisfaction (SAPS) which uses a 5 point scale (0–4) for responses. The continuous score range for SAPS is 0 to 28. Categorical responses for SAPS are defined as 0–10 very dissatisfied, 11–18 dissatisfied, 19–26 satisfied, and 27–28 very satisfied. We used linear regression analysis to examine the associations between patients’ characteristics and their satisfaction with the Thrombosis Service.

**Results:**

Of the 1058 surveys distributed, 563 were returned. The mean score for the SAPS was 22.1 (SD 4.1, range 8 to 28). For the categorical response, 85% were satisfied or very satisfied with the Thrombosis Service. The multivariate analysis showed patients with post-secondary education were more satisfied with the Thrombosis Service (β-coefficient 1.6153, *p* = 0.024), and patients taking warfarin were less likely to be satisfied with the Thrombosis Service (β-coefficient -1.5832, *p* = 0.0390).

**Conclusions:**

The majority of survey participants (85%) who attended an appointment in one of the Thrombosis Service clinics were satisfied or very satisfied with the care they received. This information may benefit other centres who are interested in developing a program to manage thrombosis and anticoagulation.

## Background

Consideration of patient satisfaction and patient reported experiences are becoming more important in the provision of health care [1]. The move towards patient centred care helps to align health services delivery with what is important to patients. In Canada, there is increased emphasis within health care organizations on providing patient centered care to address patient’s needs and improve quality care [2]. Tools such as the Canadian Patient Experiences Survey on Inpatient Care are being used to meet accreditation requirements [3]. Assessing patient satisfaction and patient experience is particularly relevant when initiating a new clinical service, to determine if the care provided meets the needs of patients.

To improve thromboembolism (TE) patient care, in October 2017 we opened a multidisciplinary Adult Outpatient Thrombosis Service (Thrombosis Service) in Newfoundland and Labrador’s largest health authority. Prior to the implementation of the Thrombosis Service care for patients with TE was provided in a fragmented manner, with long wait times for specialist care. The Thrombosis Service is designed to provide the continuum of evidence-based care required by this patient population, from the initial treatment of a new TE (with a focus on venous thromboembolism, VTE) event to long term management. Patient care is provided by a specialized multidisciplinary team. The Thrombosis Service is a comprehensive thrombosis and anticoagulation management program with unique, interrelated clinics. The service includes an Emergency Thrombosis Clinic for care post an acute episode of venous thromboembolism; Thrombosis Clinics addressing general thrombosis or anticoagulation related questions and follow up; Anticoagulation Management Clinics for management of long-term anticoagulation; and a Perioperative Anticoagulation Management Clinic for patients receiving anticoagulation requiring surgery or procedures. The Thrombosis Service is staffed during weekdays by full and part-time clinical pharmacists, a Medical Director (Thrombosis Physician/Hematologist), additional hematologist support as needed, and clerical support. The service model is designed with pharmacists as the first point of patient contact. A pharmacist completes the initial patient assessment, presents the case for discussion with the Thrombosis physician/hematologist, provides patient-centred education on medication therapy, and facilitates medication access and insurance coverage as required. Patients on long-term anticoagulation are followed up in pharmacist-led clinics. Pharmacists also respond to patient questions regarding their anticoagulation management, as well as healthcare providers’ questions regarding the Thrombosis Service.

A comprehensive evaluation of the Thrombosis Service included an assessment of patient satisfaction with the model of care. The use of self-reported measurement instruments help gather information about patient satisfaction and experience of care and are a key element in the evaluation and monitoring of quality of care [2]. Previous published surveys have primarily assessed patient satisfaction with anticoagulation management services for specific medications, such as warfarin [4–9]. We did not identify any studies assessing patient satisfaction with a comprehensive multidisciplinary Thrombosis Service. The objective of the cross-sectional survey was to assess patient satisfaction with the care received from the Thrombosis Service.

## Methods

### Study design and period

The study employed a cross-sectional study design and included all patients who had attended at least one appointment with the Thrombosis Service between October 10, 2017 and May 31, 2019.

### Setting

The Thrombosis Service is located within the Eastern Region Health Authority, the largest integrated health authority in Newfoundland and Labrador (NL). The authority offers the full continuum of health and community services including public health, long-term care, community services, hospital care and unique provincial programs and services, and serves a population of over 300,000, and is responsible for a number of unique provincial programs.

### Patient population

Patients were eligible for the study if they were 18 years of age or older and had a valid mailing address within the hospital records. Patients were identified through clinic records available in the Health Authority’s electronic medical record. Patients were excluded if on review of the hospital electronic medical record (EMR) they were identified as deceased.

### Survey method and data collection

Patients were mailed an anonymous survey in October 2019, available in English, which included a cover letter and return stamped envelope. The cover letter indicated for patients to complete the survey to specifically rate their visit with the Thrombosis Service. A follow-up reminder letter and survey was mailed about 2 weeks after the first survey [10]. Data were collected on the following parameters: (1) patient demographic characteristics (2) anticoagulation therapy; (3) satisfaction with the Thrombosis Service evaluated by the Short Assessment of Patient Satisfaction (SAPS) instrument [11]. We had performed a literature review to identify a validated, short, easily completed paper-based survey tool that was applicable to our patient population and captured important domains of patient satisfaction. The SAPS is a generic measure of patient satisfaction comprised of a short seven-item scale that have 5-point responses, scored as 0 to 4 [11]. The SAPS assesses the core domains of patient satisfaction that includes treatment satisfaction, explanation of treatment results, clinician care, participation in medical decision-making, respect by the clinician, time with the clinician, and satisfaction with clinic care. The SAPS scale was noted to exceed the Loevinger H criteria for a strong unidimensional scale, and had a Cronbach α = 0.86 [12, 13]. Permission from the instrument authors was obtained for use of the SAPS, and the seven-item survey was used as it appeared in the original publication, with “doctor/pharmacist” used for the option of “Doctor/other health professional” in the original survey. The score ranges from 0 (extremely dissatisfied) to 28 (extremely satisfied), as a continuous score. Additionally, categorical scores are defined as 0–10 very dissatisfied, 11–18 dissatisfied, 19–26 satisfied, and 27–28 very satisfied.

### Data analysis

The primary outcome was satisfaction with the care received at the Thrombosis Service as measured by the seven-item SAPS scale. Multiple imputation was used to account for up to two missing responses to the SAPS items and surveys missing more than three SAPS responses were dropped from data analysis, as recommended by the survey authors*.* Descriptive statistics were used to characterize study participants–frequencies and percentages for categorical and means with standard deviations (SDs) for continuous variables. All study variables (demographic and assessed) were categorical except patient satisfaction score. We used the univariate linear regression and analysis of variance (ANOVA) to test for statistical differences between demographic variables–age, sex, level of education, anticoagulant use and duration–and patient satisfaction score as continuous variables. Finally, multiple linear regression was used to assess the effects of socio-demographic characteristics of the study participants on overall satisfaction with the Thrombosis Service to determine the predictors of satisfaction after controlling or adjusting for all other variables in a single model. Several approaches were used to test assumptions underlying linear regression modelling. Residuals plots were examined to ensure the assumptions underlying linear regressions analysis are met. In all analyses, *P* < 0.05 was considered statistically significant. All statistical analyses were performed using Statistical Package for the Social Sciences (SPSS) (Version 26).

## Results

Of 1058 eligible patients mailed surveys, 563 were returned, providing a response rate of 52.5%. Of the 563 returned surveys, nine were excluded due to more than two SAPS items with missing data. Patients’ characteristics are reported in Table 1.

**Table 1:** Patients’ descriptive sociodemographic characteristics

Characteristics	N (%) (*n* = 554^a^)
Sex	
Male	282 (51.1)
Female	269 (48.7)
Other	1 (0.2)
Age (Years)	
< 30	4 (0.7)
30—49	67 (12.0)
50—69	270 (48.9)
> 70	211 (38.2)
Education	
Less than high school	109 (19.8)
High school diploma	73 (13.3)
Post Secondary School	368 (66.9)
Anticoagulant medication use	
Direct Oral Anticoagulant (DOAC)	296 (54.1)
Warfarin	102 (18.6)
Low Molecular Weight Heparin (LMWH)	27 (4.9)
Antiplatelet	18 (3.3)
No anticoagulant	104 (19.0)
Anticoagulant Duration (years)	
< 1	152 (27.9)
1–5	255 (46.9)
> 5	137 (25.2)

^a^Not all sections total 554 as not all patients answered all sociodemographic survey questions

The mean score and standard deviation for patients’ satisfaction with the Thrombosis Service as measured by the SAPS tool was 22.1 (SD 4.1, range 8.0–28.0). Categorical scores for the SAPS tool showed that 18.1% (*n* = 100) of respondents were very satisfied, 67.0% (*n* = 371) were satisfied, 13.7% (*n* = 76) were dissatisfied, and 1.3% (*n* = 7) were very dissatisfied. Overall, 85% of patients reported a level of satisfied or very satisfied with the Thrombosis Service.

Patient characteristics were evaluated for associations with satisfaction with the Thrombosis Service in univariate analysis (Table 2). We found that post-secondary education (β = 2.2111, *p* = 0.001) was associated with improved satisfaction with the Thrombosis Service, whereas anticoagulant use with warfarin (β = -1.7891, *p* = 0.013) and longer duration of anticoagulation (1 to 5 years, β = -1.4472, *p* = 0.026; more than 5 years, β = -1.9476, *p* = 0.009) were associated with lower satisfaction with the Thrombosis Service.

**Table 2:** Univariate analysis by sociodemographic characteristics of study participants

Variables	Patient Satisfaction Score		
	β-Coefficient	Mean(± SD)	*P-value*
Age (years)			
< 30	reference	24.0 (2.6)	
30—49	-2.653	22.2 (4.6)	0.416
50—69	-2.226	22.5 (3.9)	0.486
≥ 70	-3.956	21.4 (3.9)	0.217
Sex			
Male	reference	21.9 (4.2)	
Female	0.4656	22.2 (3.9)	0.392
Education Level			
Less than High School	reference	21.1 (4.2)	
Secondary School	0.3498	21.4 (3.7)	0.712
Post-secondary	2.2111	22.9 (4.5)	0.001
Anticoagulant Use			
DOAC	reference	22.3 (3.8)	
Warfarin	-1.7981	21.1 (4.3)	0.013
LMWH	-0.5579	21.9 (3.9)	0.668
Antiplatelet	-1.5050	21.4 (3.5)	0.323
No anticoagulant	1.0364	22.9 (4.2)	0.147
Anticoagulant Duration (years)			
Less than 1 year	reference	22.8 (4.1)	
1 – 5 years	-1.4472	21.9 (3.9)	0.026
More than 5 years	-1.9476	21.6 (3.9)	0.009

After adjusting for all other demographic characteristics in multivariate regression analysis, only post-secondary education (β = 1.6153, *p* = 0.024) and anticoagulant use with warfarin (β = -1.5832, *p* = 0.039) were identified as significant predictors of satisfaction with the Thrombosis Service (Table 3). Patients with post-secondary education were more likely to be satisfied with the Thrombosis Service, whereas patients taking warfarin as their anticoagulant were less likely to be satisfied with the Thrombosis Service. The SAPs tool demonstrated a high internal consistency when assessed for reliability using Cronbach’s alpha (0.62) and Composite reliability tests (0.86).

**Table 3:** Multivariate analysis by sociodemographic characteristics of study participants

Variables	Patient Satisfaction Score	
	β-Coefficient	*P-value*
R^2^	0.026	0.011
Age (years)		
< 30	reference	
30- 49	-1.8664	0.562
50–69	-0.9344	0.769
≥ 70	-2.0030	0.531
Sex		
Male	reference	
Female	0.0530	0.924
Education Level		
Less than high school	reference	
Secondary school	0.0192	0.984
Post-secondary education	1.6153	0.024
Anticoagulant Use		
DOAC	reference	
Warfarin	-1.5832	0.039
LMWH	-0.8719	0.490
Antiplatelet	-0.9894	0.519
No anticoagulant	0.7046	0.399
Anticoagulant Duration (Years)		
Less than 1 year	reference	
1 – 5 years	-0.5575	0.442
More than 5 years	-0.60588	0.484

## Discussion

The majority of survey respondents (85%) who attended an appointment in one of the Thrombosis Service clinics were satisfied or very satisfied with the care they received. Most of the respondents were 50 years or older (88%), reflective of patients with conditions requiring thrombosis and anticoagulation management. Patients with post-secondary education were more likely to be satisfied with their care, whereas patients receiving warfarin as their anticoagulant were less likely to be satisfied with their care.

Studies that have measured patient satisfaction related to thrombosis care have shown similar satisfaction results to this study, however the tools used have varied and may not have been validated. Webb et al. examined patient satisfaction with VTE treatment across multiple health care settings via the use of patient self-completed online survey [14]. As part of the survey, patients indicated their overall level of satisfaction with the provider who provided the majority of their VTE care. Of an eligible sample of 1000, 907 completed the survey. Most patients (87.2%) were satisfied or very satisfied with their VTE care experience across all health care settings, and 74.9% were satisfied or very satisfied with their VTE care when provided by specialized thrombosis services. Zed et al. evaluated the efficacy, safety and patient satisfaction of a pharmacist-managed, emergency department based outpatient treatment for VTE disease [15]. Of the 305 patients, 231 returned the survey, and 96.9% of the patients were very satisfied or satisfied with the treatment they received in the outpatient DVT program.

In our study, patients with post-secondary education were more likely to be satisfied with their care. A number of patient factors, including patient age, income, education and comorbidity, may influence patient satisfaction [16]. A systematic review of determinants of patient satisfaction showed inconsistent results of the strength and direction of associations with patient factors, including level of education [17]. Webb et al. showed no difference in patient demographics and level of satisfaction with overall VTE care [14]. Given that there are inconsistent results for the influence of post-secondary education on patient satisfaction, our result of increased satisfaction may not be reliable.

Patients in our study receiving warfarin as their anticoagulant were less likely to be satisfied with their care, however our study was not designed to probe additional details regarding specific anticoagulant therapy. Published studies examining satisfaction of patients receiving warfarin versus DOACs have shown those receiving warfarin are less satisfied with their anticoagulant therapy [8, 9, 18], particularly if they have switched from warfarin to a DOAC [8]. Improved patient convenience, reduced frequency of medical contact, and fewer side effects have been noted as reasons why patients are more satisfied with DOACS [8].

Future study should explore the areas where patients indicated they were dissatisfied to assess the reasons for, and possible initiatives to address the dissatisfaction. Patients receiving warfarin as their anticoagulant medication were less likely to be satisfied with their care, and the specific reasons for this in our patient population should be explored further. Our study primarily assessed the patient satisfaction with the Thrombosis Service; future work could also include measurement of patient experience to more fully assess the quality of care from a patient’s viewpoint [2].

Patient satisfaction studies have been criticized for lack of standardized or validated measures, as well as the potential inaccurate perception of patient satisfaction as a measure for quality care [19]. The terms patient satisfaction and patient experience are at times used interchangeably in the literature [20], however may not refer to the same measure, leading to confusion. A recent publication provided a framework for person-centered measures of health system quality and responsiveness, and provided more clear definitions: patient satisfaction is the patients’ evaluation of the care provided relative to their expectations (an outcome measure), and patient experience is the interactions patients have with the health care system (process indicator) [21]. The two terms can been seen as related, with patient experience of care influencing patients satisfaction with care. Defining the purpose of the measurement (improving quality of care and/or system accountability) will help define which measurement to use, with a potential role for both types of data.

Satisfaction surveys are noted to have a number of biases. Social-desirability bias may cause respondents to answer questions in a manner that will be viewed favorably by others, and could overestimate satisfaction. However, strategies used to minimize social desirability biases in our study included the use of anonymous surveys, as well as the use of researchers not involved in direct patient care to coordinate and collect data. Acquiescence bias is the tendency of respondents to agree to the statement they are presented, regardless of its content [22]. Thus surveys with all positively framed questions may result in a high satisfaction rating. The SAPS survey addresses this through having a combination of positive and negative worded statements.

### Study limitations

Given that this was an observational, retrospective cross-sectional study, causal inferences cannot be made. Additionally, our study may have been subject to recall bias, as some patients may have difficulty remembering the details of their encounter, particularly if there was a delay between the clinical encounter and the survey receipt. The use of SAPS may overestimate/underestimate responses owing to social-desirability bias common to self-reported instruments. However, we adopted strategies to reduce social desirability bias. First, surveys were anonymous to avoid pressure on participants to respond in a socially acceptable way. Secondly, researchers involved in direct patient care at the TS were neither involved in data collection nor coordination of the survey but only contributed to the preparation of the manuscript. This was to reduce any impact healthcare provider–patient relationships might have on participants’ responses to the survey.

It was a single-center study based on one model of care, which may limit its generalizability. Just under half (47.5%) of patients did not respond to the survey, and it is unknown if this would change the results. However, previous data showed that over an approximately seven and half month period, patients attending the Thrombosis Service had an average age of between 55 and 65 years, and 40 to 57% were male, which is similar to the population in this study [23]. As this was intended to be an anonymous survey, we did not link a survey to patient specific data, e.g., number of clinic visits, time since attendance at clinic, which limited the possible analysis.

## Conclusion

This is the first study that used the SAPS to assess patient satisfaction with a multidisciplinary Thrombosis Service. Overall, patients were satisfied with the care received at the Thrombosis Service, as indicated by the survey scores. This information may benefit other centres who are interested in developing a program to manage thrombosis and anticoagulation. Future study should explore the areas where patients indicated they were dissatisfied, as well as details of patient experiences.

## Data Availability

The datasets used and/or analyzed during the current study are not publicly available given that participants consented for their data to be available only to researchers of this study, but may be available from the corresponding author on reasonable request.
